# A case report of simple mucinous cyst of pancreas detected by rupture

**DOI:** 10.1016/j.ijscr.2021.106468

**Published:** 2021-10-02

**Authors:** Naoko Sekiguchi, Shinsuke Nakashima, Yujiro Tsuda, Jin Matsuyama, Masakazu Ikenaga, Terumasa Yamada

**Affiliations:** Department of Surgery, Higashiosaka City Medical Center, Nishiiwata 3-4-5, Higashiosaka, Osaka 567-8588, Japan

**Keywords:** Simple mucinous cyst, Rupture, Pancreas, Case report

## Abstract

**Introduction:**

Simple mucinous cyst (SMC) of pancreas is a disease defined by the Baltimore Consensus in 2014. Pancreatic mucus-producing neoplasms are considered to be premalignant tumors, but SMC is not considered to have a risk of malignancy or recurrence.

**Presentation of case:**

The case was a woman in her 50s with a chief complaint of abdominal pain. A blood exam showed an increase in the inflammatory response, and a slight increase of Amylase. CT showed a cystic lesion 80 mm in size at tail of the pancreas, and disproportionate fat stranding and ascites around it. We diagnosed peritonitis associated with the rupture of a cystic lesion accompanied by pancreatitis. Abdominal pain was improving, and we decided to proceed with the detailed examination. MRI showed a uniform hyper-intensity on T2WI, and a nodular-like hypo-intensity was observed inside, which was enhanced. During the follow-up, the lesion had gradually grown and re-ruptured. As we could not deny malignancy by image findings, distal pancreatectomy was performed. The intracystic fluid was browny and turbid, and Amylase, CEA and CA19-9 of the cystic fluid were elevated. We diagnosed it SMC by histopathological findings. Currently, she had no recurrence for 1 year.

**Discussion:**

SMC is a type of true cysts, so rupture was rare. However, if the cyst wall becomes weak due to complications such as acute pancreatitis. It is probable that our case had pancreatitis and the cyst wall was weakened.

**Conclusion:**

SMC detected by rupture was very rare, so we report this case.

## Highlights

• SMC of the pancreas in which surgery was required due to rupture was rare case.
• This is unique that it looked like an nodule due to the gathering of cystic walls.
• Non-neoplastic lesions should be considered if nodule grow and shrink over time.

## 1 Introduction

Simple mucinous cyst (SMC) of pancreas is a disease defined by the Baltimore Consensus in 2014 [1]. Pancreatic mucus-producing neoplasms, for example intraductal papillary mucinous neoplasms (IPMN) and mucinous cystic neoplasms (MCN), are considered to be premalignant tumors, but SMC is not considered to have a risk of malignancy or recurrence. We report a rare case of SMC of the pancreas in which surgery was required due to rupture and the possibility of malignancy.

This report has been reported in line with the SCARE criteria [2].

## 2 Case presentation

A 50-year-old female presented with sudden abdominal pain. Abdominal computed tomography (CT) was performed and revealed a pancreatic cystic lesion. So, she was referred to our hospital for detail examination of pancreatic cystic lesion. On physical examination, there were tenderness and spontaneous pain on her entire abdomen. She had been treated for acute pancreatitis twice and didn't have any medical and family history. Blood examination showed increasing inflammatory response, WBC 10180/μl and CRP 23.6 mg/dl, and tumor markers, CEA, CA19-9 and CA125, were not elevated.

**Fig. 1:**
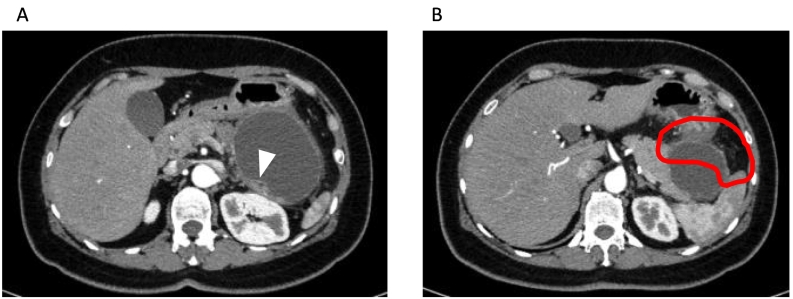
A/B; CT. The contrast-enhanced CT revealed a unilocular cystic lesion in pancreatic tail sized in 80 mm which had partial thicken wall (arrow head). There were disproportionate fat stranding around cystic lesion (in circle).

**Fig. 2:**
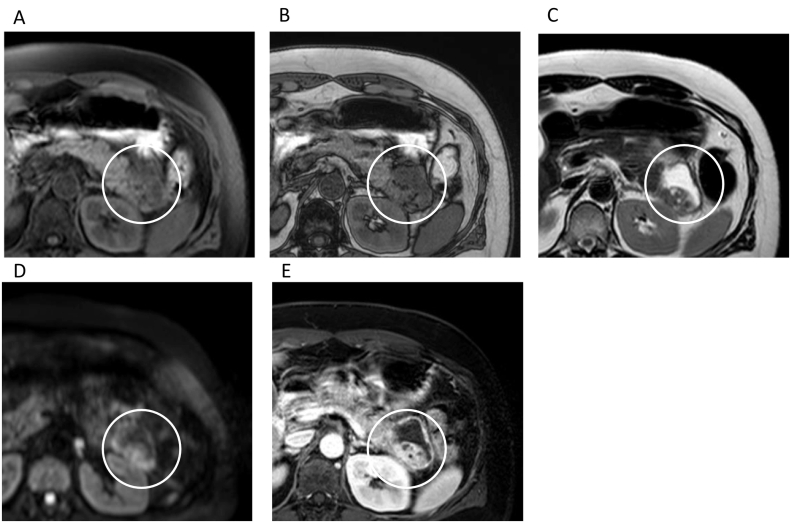
A; T1WI in-phase, B; T1WI opposed-phase, C; T2WI, D; DWI, E; Dynamic early phase. MRI showed the lesion had a solid component which was low intensity in T1WI, high intensity in T2WI and DWI. The nodule was enhanced in the early phase, and had microcystic lesions inside.

**Fig. 3:**
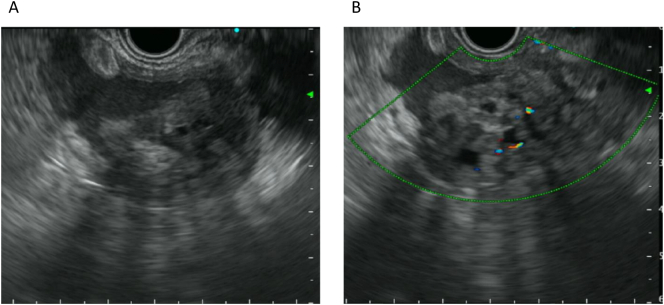
A; EUS, B; EUS with collor doppler imaging. EUS showed that the nodule was protruding into the lumen and had blood flow.

**Fig. 4:**
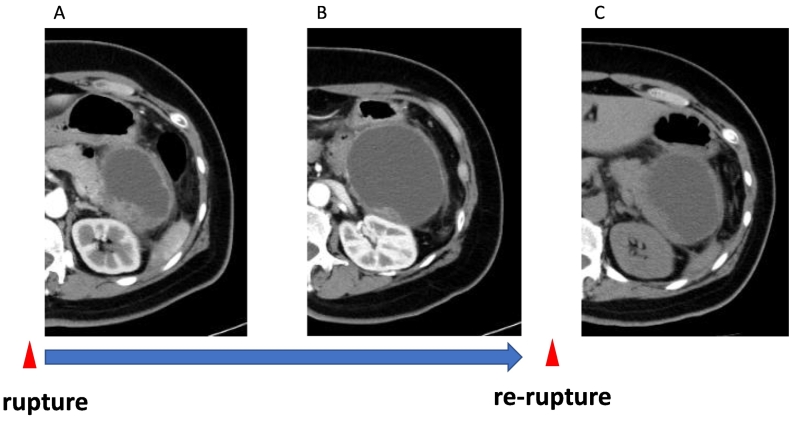
A; CT at first visit, B; one month after, C; after re-rupture. At first visit, the cyst was 80 mm in size and had solid conponents inside. One month after, the cystic lesion had gradually grown to 96 mm and solid components became slightly smaller. 30 days after the follow up CT, she presented with sudden abdominal pain. We diagnosed with re-rupture because of the shrinkage of the cystic lesion.

The contrast-enhanced CT revealed a unilocular cystic lesion in pancreatic tail sized in 80 mm which had partial thicken wall. There were disproportionate fat stranding around cystic lesion (Fig. 1), and ascites in the Douglas fossa. Based on the image findings, we suggested that she had diffuse peritonitis due to rupture of a pancreatic cyst with pancreatitis. We decided to treat pancreatitis and proceed with the detail examination of pancreatic cystic lesion because her symptoms were improving over time. MRI showed that the lesion was low to moderate intensity in T1-weighted image and high intensity in T2-weighted image. The lesion had a nodule-like low intensity area inside. The nodule enhanced in the early phase, and had high intensity area in diffusion-weighted images (Fig. 2). In MRCP, the communication to the main pancreatic duct was not revealed. EUS showed that the nodule was protruding into the lumen and nourished by the splenic artery (Fig. 3). One month after the first visit, the cystic lesion had gradually grown to 96 mm (Fig. 4). 30 days after the follow up CT, she presented with sudden abdominal pain and admitted to our hospital again. We diagnosed with re-rupture because of the shrinkage of the cystic lesion and presence of ascites around the lesion. Pseudopancreatic cyst was also considered because of the complication and past history of pancreatitis, but we could not deny neoplastic disease because MRI and EUS showed a solid component with blood flow in the cystic lesion. Since there was a history of rupture, it was unlikely that a tumor with a thick capsule like MCN would develop. We suspected SPN (Solid Pseudopapillary Neoplasm) first because of pancreatic tail development at a relatively young age, so we performed distal pancreatectomy.

The operation was performed by specialists of pancreatic resection who had been in practice for over 10 years. In operative findings, there were adhesions due to repeated ruptures around the cyst lesion. The cystic lesion surface was smooth, and cysts contained fluid. We punctured the intra-cystic fluid and collected a brownish fluid. The biochemical examination of the intracystic fluid was Amylase 27,000 U/l, carcinoembryonic antigen 280 ng/dl, and carbohydrate antigen 7900 U/ml by biochemical examination. The operation time was 4 h 19 min, and the blood loss was 1400 ml.

**Fig. 5:**
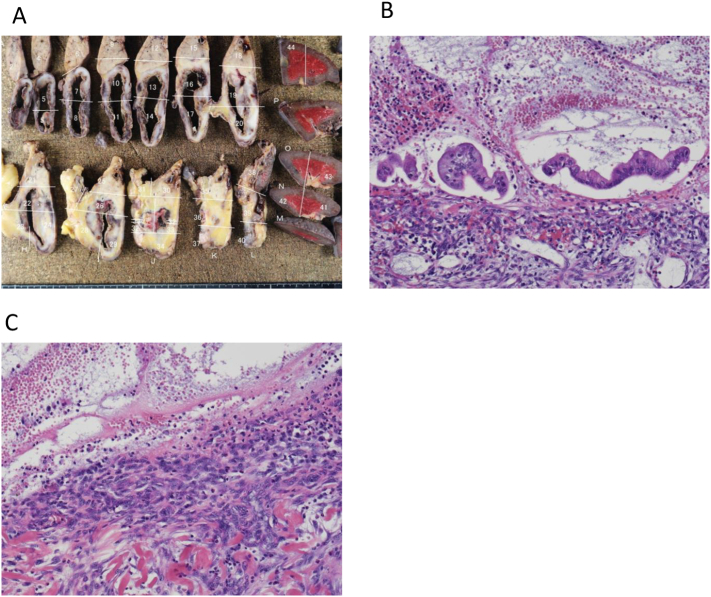
A; resected specimen. The cyst had no solid components inside. B; HE stain (×100). Most cells have fallen off due to inflammation, but some are covered with simple columnar epithlium cells. C; HE stain (×100). The cyst wall had degenerative changes, including granulation-like tissue, hyalinization, myxoid stroma and calcification.

The pathological findings revealed that there were no nodules, and mucinous cells covered inside the cyst. Immunostaining was performed, but no ovarian type stroma characteristic of MCN was found. The cells were poorly atypical and no neoplastic growth was observed, and granulation tissue was growing in the stroma (Fig. 5). Finally, we diagnosed Simple Mucinous Cyst (SMC).

The course after the operation was uneventful. She discharged after 32 days after surgery. She had lived for a year after surgery without any evidence of metastasis and recurrence.

## 3 Discussion

Two German pathologists, Kosmahl and Klöppel, and their colleagues in 2002 were the first to describe this type of mucinous cyst in the English-language literature [3]. According to the 2014 Baltimore Consensus Meeting, simple mucinous cysts are defined as macroscopic cysts that are greater than 1 cm in size with gastric-type flat mucinous lining and minimal cytologic atypia without ovarian-type stroma [1].

SMC has been reported as 2.1– 3.4% in resected pancreatic lesions [4], [5], and occurs more commonly in female (2.8:1 female to male ratio) with a mean age at diagnosis of 64 years [6]. As a result of searching with keywords, simple mucinous cyst and pancreas, in PubMed, only one case was reported by cystic rupture. The case had penetrated the colon because of an intracystic infection [7]. SMC is a type of true cysts lined by mucus producing cells, so rupture was rare. However, if the cyst wall becomes weak due to complications such as acute pancreatitis, it is considered that there is a possibility of perforation or penetration in the surrounding area. It is probable that our case had pancreatitis and the cyst wall was weakened.

Although mucinous pancreatic cystic lesions are generally considered to be precursor lesions of pancreatic adenocarcinoma owing to their common association or coexistence with cancer [8], SMC showed no evidence of recurrence or malignant transformation after resection during a mean follow-up time of 2 years [3]. However, its preoperative differentiation from mucinous neoplasms which includes MCNs or IPMNs is difficult due to several overlapping clinical, radiological, and biochemical features [9], [10]. Most cases lacked communication with the pancreatic duct, but 7% have communication. These image findings also raised the possibility of IPMN or retention cyst, and it makes difficult to discrimination from other pancreatic cystic lesion.

In this case, we couldn't deny malignant potential because preoperative image findings showed the cystic lesion had solid components with blood flow. But histopathological findings revealed no nodule inside the cyst. When viewed retrospectively, nodular components were found inside the cyst at the first rupture, but the larger the cyst grew, the smaller the nodular components became in the follow-up CT. So, it seems that the cyst wall was gathered and looked like a nodule due to the shrinkage of the cyst due to the rupture. Intracystic nodules generally mean to be malignant in cystic pancreatic lesions, but if the nodules grow and shrink over time, it is necessary to consider the possibility like this case.

Past report showed that analysis of cystic fluid may be useful in benign and malignant cysts. CEA level, CA19-9 level and K-RAS mutation are more frequently observed premalignant cystic lesion [11]. 82% of SMC had elevated cyst fluid CEA levels [12]. In our case, in addition to the increased CEA level in the intracystic fluid, the CA19-9 level was also elevated. Recent reports revealed that SMC have a median of 4 mutations per cyst, such as K-RAS and BRAF. These molecular studies support a neoplastic process, so the malignant potential of SMC is still debatable.

## 4 Conclusion

We experienced a rare and unique case of ruptured SMC which cyst wall was gathered and looked like a intracystic nodule due to the shrinkage of the cyst. Since there is no long-term observational study of SMC and it has one aspect of neoplastic lesions, follow-up is considered necessary in the future.

## Abbreviations

CT: Computed tomography
IPMN: Intraductal papillary mucinous neoplasm
MCN: Mucinous cystic neoplasm
MRI: Magnetic resonance imaging
T1WI: T1 weighted image
T2WI: T2 weighted image
DWI: diffused weighted image

## Ethical approval

This study of case report is exempt from ethnical approval in ethics committee of our institution.

## Funding

None.

## CRediT authorship contribution statement

Clinical treatment: Naoko Sekiguchi, Shinsuke Nakashima, Masahiro Koh, Masami Ueda, Yujiro Tsuda, Tsukasa Tanida, Jin Matsuyama, Masakazu Ikenaga and Terumasa Yamada

Collected data: Naoko Sekiguchi, Shinsuke Nakashima

Assessment and discussion: Naoko Sekiguchi, Shinsuke Nakashima and Terumasa Yamada

Wrote the paper: Naoko Sekiguchi, Shinsuke Nakashima and Terumasa Yamada

## Guarantor

Corresponding author, Terumasa Yamada

## Research registration

None.

## Consent

Informed consent has been obtained from the patient and all identifying details have been omitted. A copy of the written consent is available for review by the Editor-in-Chief of this journal on request.

## Provenance and peer review

Not commissioned, externally peer-reviewed.

## Declaration of competing interest

None.
